# Normal Evoked Response to Rapid Sequences of Tactile Pulses in Autism Spectrum Disorders

**DOI:** 10.3389/fnhum.2016.00433

**Published:** 2016-09-16

**Authors:** Santosh Ganesan, Sheraz Khan, Keri-Lee A. Garel, Matti S. Hämäläinen, Tal Kenet

**Affiliations:** ^1^Department of Neurology, Massachusetts General HospitalBoston, MA, USA; ^2^Athinoula A. Martinos Center for Biomedical Imaging, Massachusetts General Hospital/Massachusetts Institute of Technology/HarvardBoston, MA, USA; ^3^Harvard Medical SchoolBoston, MA, USA; ^4^McGovern Institute for Brain Research, Massachusetts Institute of TechnologyCambridge, MA, USA; ^5^Department of Radiology, Massachusetts General HospitalBoston, MA, USA

**Keywords:** somatosensory, cortex, MEG, autism spectrum disorder

## Abstract

Autism spectrum disorder (ASD) is a developmental disorder diagnosed behaviorally, with many documented neurophysiological abnormalities in cortical response properties. While abnormal sensory processing is not considered core to the disorder, most ASD individuals report sensory processing abnormalities. Yet, the neurophysiological correlates of these abnormalities have not been fully mapped. In the auditory domain, studies have shown that cortical responses in the early auditory cortex in ASD are abnormal in multiple ways. In particular, it has been shown that individuals with ASD have abnormal cortical auditory evoked responses to rapid, but not slow, sequences of tones. In parallel, there is substantial evidence of somatosensory processing abnormalities in ASD, including in the temporal domain. Here, we tested the somatosensory domain in ASD for abnormalities in rapid processing of tactile pulses, to determine whether abnormalities there parallel those observed in the auditory domain. Specifically, we tested the somatosensory cortex response to a sequence of two tactile pulses with different (short and long) temporal separation. We analyzed the responses in cortical space, in primary somatosensory cortex. As expected, we found no group difference in the evoked response to pulses with long (700 ms) temporal separation. Contrary to findings in the auditory domain, we also found no group differences in the evoked responses to the sequence with a short (200 ms) temporal separation. These results suggest that rapid temporal processing deficits in ASD are not generalized across multiple sensory domains, and are unlikely to underlie the behavioral somatosensory abnormalities observed in ASD.

## Introduction

Autism spectrum disorder (ASD) is diagnosed behaviorally, with core abnormalities in the social interactions, communication, and repetitive behavior domains. While not core to ASD by definition, it is well known that ASD individuals experience many sensory abnormalities, with both sensory hypo- and hyper-sensitivities documented in most individuals with ASD (Leekam et al., 2007; Tomchek and Dunn, 2007; Cascio et al., 2008). Despite the high prevalence of such abnormalities in ASD, studies investigating the neural correlates of these abnormalities are under-represented relative to studies of the neural correlates of social abnormalities in ASD for instance. Within the sensory modalities, in turn, studies of abnormal somatosensory processing are under-represented relative to studies of abnormal visual or auditory processing in ASD (Marco et al., 2011).

Abnormalities in sensory processing might arise from many different sources. One line of investigation centers on temporal processing deficits, i.e., deficits caused by abnormal processing of stimuli in time. This idea first arose in the context of dyslexia and language deficits (Benasich and Tallal, 2002). Since the processing of speech sounds requires very rapid analysis of incoming sounds often separated by no more than a few milliseconds, abnormally slow processing in the auditory domain would result in behavioral deficits with the processing of speech sounds, and therefore language. While this idea has been explored primarily in language impaired populations, it has also been explored in ASD. Specifically, it was found that ASD individuals indeed showed deficits in rapid temporal processing in the response to a rapid sequence of two tone sounds, spaced 150 ms apart (Oram Cardy et al., 2005). While that particular study found similar deficits in individuals with language impairment, this result was qualitative rather than quantitative, and the extent to which such a deficit might contribute to ASD remains unexplored. Other studies of temporal processing and temporal integration deficits in ASD, not using the rapid presentation paradigm, found deficits that do appear to correlate with ASD features more specifically (e.g., McPartland et al., 2004; Foss-Feig et al., 2010; Roberts et al., 2011).

While somatosensory processing does not rely on rapid processing in the same way language does, temporal accuracy is nonetheless crucial for somatosensory processing, and different behavioral profiles are associated with different frequencies of somatosensory stimulation (Tannan et al., 2005; Francisco et al., 2011). Investigating the profile of cortical somatosensory responses in ASD is important because of the abundant behavioral evidence showing abnormal somatosensory processing in ASD, indicating profiles of hypo- and hyper-sensitivity to tactile stimulation (Tomchek and Dunn, 2007; Wiggins et al., 2009; Abu-Dahab et al., 2013). Neurophysiologically, the findings are somewhat mixed. At least one study found enhanced responses to vibrations and thermal pain in ASD (Cascio et al., 2008), but reduced responses have also been documented in the somatosensory domain in ASD (Marco et al., 2012; Coskun et al., 2013; Khan et al., 2015). Prior work on rapid temporal processing in ASD found no differences in ASD in response to tactile taps spaced 330 ms apart (Marco et al., 2012). However, whether this is true for even more rapid presentations of tactile taps remains unknown. Given the mixed findings in the field, showing increased, reduced, or normal cortical responses to somatosensory stimuli in ASD, we sought to investigate and further elucidate whether a deficit in rapid temporal processing might also underlie some of the somatosensory processing abnormalities observed in ASD. To that end, we used magnetoencephalography (MEG) to measure the evoked responses to rapid (200 ms interstimulus interval) versus slow (700 ms interstimulus interval) sequences of two somatosensory pulses applied to the fingertips, in individuals with and without ASD. We hypothesized that on this faster time interval than previously measured (Marco et al., 2012), like in the auditory domain (Oram Cardy et al., 2005), the ASD groups will be characterized by reduced responses to the second of the two pulses in the rapid sequence, but not in the slow sequence.

## Materials and methods

### Participants

Participants were 12 children with ASD, ages 6–21, and 22 Typically Developing (TD) children, ages 7–21. ASD participants had a prior clinically verified ASD diagnosis, met a cutoff of >15 on the Social Communication Questionnaire (SCQ), Lifetime Version, and were assessed with either Module 3 (*n* = 3) or 4 (*n* = 12) of the Autism Diagnostic Observation Schedule (ADOS, Lord et al., 1999), administered by trained research personnel who had established inter-rater reliability. Individuals with autism-related medical conditions (e.g., Fragile-X syndrome, tuberous sclerosis) and other known risk factors (e.g., premature birth) were excluded from the study. All TD participants were below threshold on the SCQ and were confirmed to be free of any neurological or psychiatric conditions, and of substance use for the past 6 months, via parent, and self-reports. All the ASD subjects were clinically diagnosed with ASD as per the DSM-5, and were considered high functioning, with both verbal and non-verbal IQ >70. The ASD and TD groups did not differ in verbal or nonverbal IQ, as measured with Differential Ability Scales (DAS). Handedness information was collected using the Dean Questionnaire (Piro, 1998). Only right-handed participants were included in the study. All the experimental protocols were approved by The Massachusetts General Hospital Institutional Review Board and all procedures were carried out in accordance with the approved guidelines. Written informed consent was obtained from all subjects. Additional details on the participants are provided in Table 1.

**Table 1:** **Behavioral measures**.

	**ASD (*n* = 12) Mean (*SD*), Range**	**TD (*n* = 22) Mean (*SD*), Range**	***P*-value**
Age	12.5 (5.21), 6–21	13.77 (3.72), 7–21	0.46
SCQ lifetime	26 (2.65), 23–28	3.13 (2.75), 0–9	0.001
SCQ current	19.4 (4.04), 14–25	5 (3.42), 1–11	0.0002
ADOS combined	12.4 (4.01), 7–19	2.33 (1.73), 0–5	*p* < 0.0001
ADOS soc	8.42 (2.68), 5–13	1.55(1.42), 0–4	*p* < 0.0001
ADOS comm	4 (1.53), 2–7	0.77 (0.83), 0–2	*p* < 0.0001
Verbal IQ	109.5 (20.07), 74–142	115.73 (13.21), 86–142	0.1
Nonverbal IQ	105.2 (19.06), 73–144	110.5 (14.06), 77–130	0.41
Touch score	57.86 (14.86), 34–76	80.73 (9.88), 60–90	0.005

*There were no significant differences between groups with respect for age, verbal, and non-verbal IQ. As expected, there were significant group differences for SCQ current and lifetime, for ADOS (combined and individual components), and for the touch score*.

### Paradigm

Participants were seated inside an MEG and two sine wave pulses of 15 psi were applied simultaneously and identically, to the index and middle right fingers, using a custom made pneumatic tactile stimulator with latex tactor tips (Briggs et al., 1998). The two adjacent fingers were stimulated simultaneously to increase the signal. The onset to peak of each pulse lasted 40 ms. Subjects were directed to sit still with arms slightly extended over an armrest, with a cloth covering placed over the arm. The two pulses with a temporal separation of either 200 or 700 ms between pulses were then delivered, with an interstimulus interval of 3 s, with a 0.5 s jitter to avoid adaptation. Stimuli timing and sequences were programmed using the psychophysics toolbox (http://www.psychtoolbox.org). A movie was presented during stimulus presentation.

### Structural MRI data acquisition and processing

A 3.0 T Siemens Trio whole body MR scanner (Siemens Medical Systems) with a 32 channel head coil was used to acquire a T1-weighted, high-resolution, magnetization-prepared rapid gradient echo structural image. Reconstruction of cortical surfaces for each subject were generated with FreeSurfer (Dale et al., 1999; Fischl et al., 1999a).

### MEG data acquisition and pre-processing

MEG data were acquired inside a magnetically shielded room (IMEDCO) using a whole-head Elekta VectorView MEG system, comprised of 306 sensors arranged in 102 triplets of two orthogonal planar gradiometers and one magnetometer. All signals were filtered between 1 and 50 Hz and sampled at 600 Hz. The position and orientation of the head with respect to the MEG sensor array was recorded continuously with help of four Head Position Indicator (HPI) coils. To allow co-registration of the MEG and MRI data, the locations of three fiduciary points (nasion and auricular points) that define a head-based coordinate system, a set of points from the head surface, and the sites of the four HPI coils were digitized using a Fastrak digitizer (Polhemus, Colchester, VT, USA) integrated with the Vectorview system. The ECG and electrooculogram (EOG) signals were recorded simultaneously to identify epochs containing heartbeats as well as vertical and horizontal eye-movement and blink artifacts. During data acquisition, on-line averages were computed from artifact-free trials to monitor data quality in real time. All off-line analysis was based on the saved raw data. In addition, 5 min of data from the room void of a subject were recorded before each experimental session for noise estimation purposes.

### Cleaning and motion correction

The data were spatially filtered using the Signal Space Separation (SSS) method (Elekta-Neuromag Maxfilter software) to suppress noise generated by sources outside the brain (Taulu et al., 2004; Taulu and Simola, 2006). This step also corrects for head motion, which is registered with 200 ms resolution, between and within runs. Cardiac and ocular artifacts were removed by signal space projection (Gramfort et al., 2013). The data were low-pass filtered at 145 Hz to remove the head position indicator (HPI) coil excitation signals.

### Epoching

The data were epoched into single trials lasting 1550 ms from 150 ms prior to stimulus onset to 1400 ms following it. A total of 50 trials per condition were collected. Epochs were rejected if the peak-to-peak amplitude during the epoch exceeded 1500 fT and 3000 fT/cm in any of the magnetometer and gradiometer channels, respectively. This resulted in the loss of 1–15 trials per participant. To maintain a constant signal to noise ratio across conditions and participants, the number of trials per condition per participant was fixed at 35, the minimum number of accepted trials that we had for each condition and participant. For conditions and participants that had more than 35 good trials, we selected the first 35 from the available trials. This results in a fixed Signal to Noise Ratio (SNR) of $\sqrt{35}$ = 5.92 with respect to the single trial for each participant. For the tactile MEG recordings, there were no group differences in the overall quality of the data, and the number of good, non-rejected trials per condition was similar between groups and across conditions. For each participant, the same set of trials was used for all analyses. Preliminary analyses of the responses in a subset of the participants for whom 45–50 trials were available showed no notable differences in the results.

### Source estimation

The cortical source space consisted of 10,242 dipoles per hemisphere, corresponding to a spacing of approximately 3 mm between adjacent source locations. The forward solution was computed using a single compartment boundary element model (Hämäläinen and Sarvas, 1989). The individual inner skull surface triangulations for this model were generated with the watershed algorithm in FreeSurfer. The current distribution was estimated using the minimum-norm estimate (MNE) by fixing the source orientation to be perpendicular to the cortex (Gramfort et al., 2014). The noise covariance matrix was estimated from data acquired in the absence of a subject prior to each session. We employed depth weighting to reduce the bias of the minimum norm estimates toward superficial currents (Lin et al., 2006). A morphing map to optimally align the cortical surface of each participant to an average cortical representation was computed in FreeSurfer (Fischl et al., 1999b). Source localization was carried out on the 35–65 ms time window, relative to stimulus onset.

### Delineating the primary somatosensory area S1

To identify S1, we first mapped the averaged MEG sensor space data to the individual cortical manifold through MNE. S1 was identified by delineating the areas that showed the maximum activation. To further quantify this area, we mapped S1 to FsAverage Space by using morphing maps, computed earlier. We also investigated responses across the entire cortical space, to identify any other areas that showed significant responses. S2 was the only other such area. For both S1 and S2, the responses were significantly more pronounced in the contralateral hemisphere, as expected. Preliminary analyses showed that the responses were not qualitatively different between S1 and S2, and therefore, for the rest of the analyses, we focused exclusively on the contralateral S1, the area with the highest SNR. Regions of interest were then delineated individually by setting a global threshold on the cortical activations at *F*-score > 8 (Dale et al., 2000; Gramfort et al., 2014). The *F*-score represented the statistical distance of cortical activation during the steady state period from the empty room MEG recordings collected before the subject arrival. Lastly, FreeSurfer labels indicated that S1 overlapped with Brodman areas 3a and 3b.

### S1 time-course

**Figure 1:**
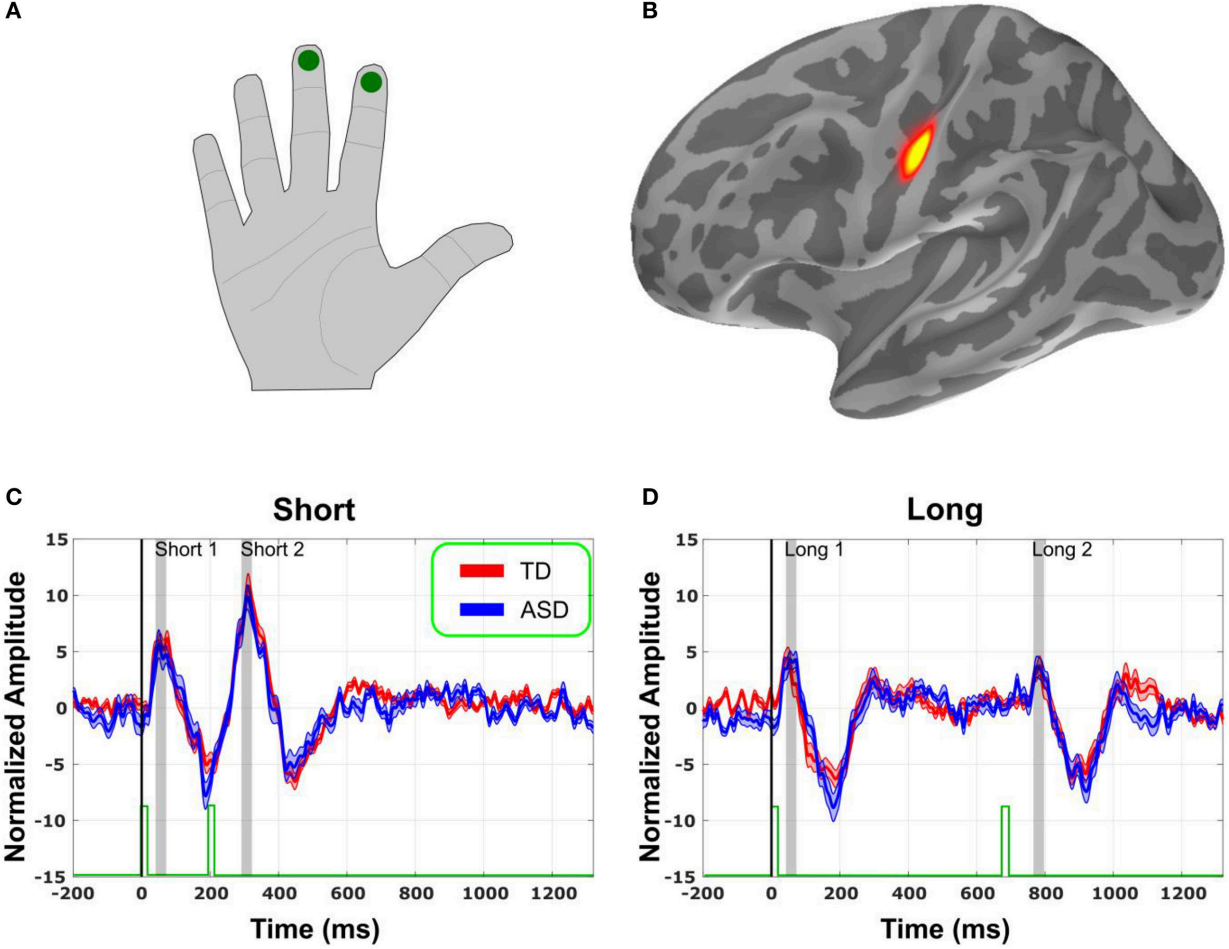
**Source localization and Evoked Responses. (A)** Schematization of location of stimulus delivery. **(B)** Evoked responses in sensory space localized to primary Somatosensory (S1) cortex (yellow). **(C)** Normalized evoked responses in S1 to tactile pulses spaced 200 ms (short) apart. Stimulus marked with green bars at the bottom. Shaded gray regions represent the time window of interest, surrounding each peak. Thirty-five to sixty-five milliseconds for peak 1 (short and long), 295–325 ms for peak 2 (short), and 760–790 ms for peak 3 (long). Shades area around the signal trace is standard error. **(D)** Same, for the stimuli spaced 700 ms (long) apart.

**Figure 2:**
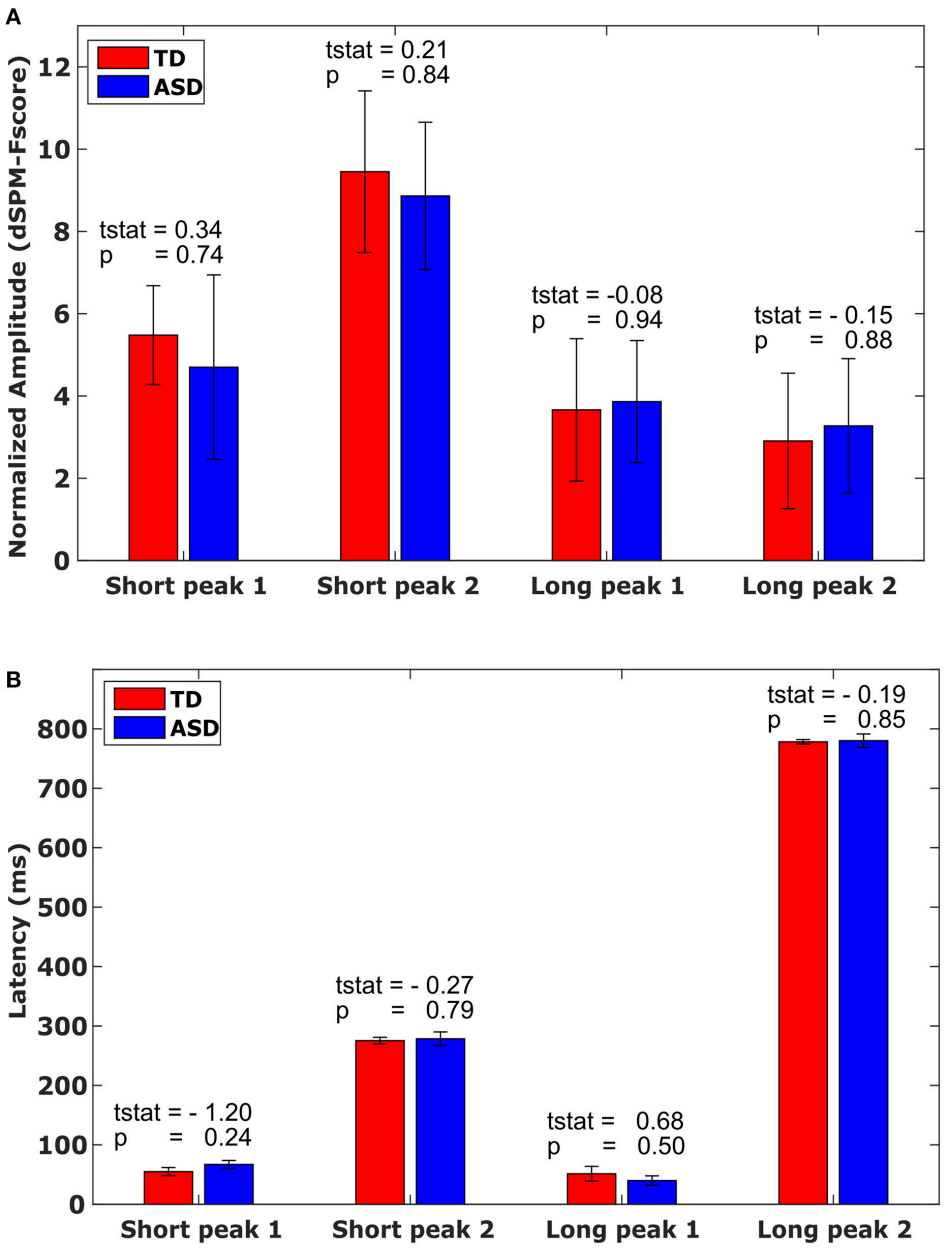
**Amplitude and Latency. (A)** Mean amplitude values with associated standard error for each M40 peak in the short and long evoked response conditions, by group, averaged across the time window marked in Figures 1C,D. **(B)** Mean latency values with associated standard error for each M40 peak in the short and long evoked response condition, by group. Bars mark the standard error.

We averaged across all vertices of the S1. This yielded a mean time-course for the S1, which was used as the seed in computations. To avoid signal cancelation, the averaging took into account the polarity mismatches that occur because of MNE estimate spreading across sources whose orientations were not aligned. This was done by flipping the polarity of the signals from sources that were oriented at >90° relative to a principal direction of the cortical normal within the S1 region. Amplitudes of the evoked responses were normalized by using the dSPM values that are *F* ratio scored with respect to the empty room recordings. It is those normalized values that are plotted on the y axis in (Figures 1C,D, 2A). Lastly, note that within the timecourse, the analysis was restricted specifically to the M40 component of the response, in order to focus on the earliest high SNR component, and thus maximize the reliability of the results. Brief analyses of later components of the response results in no discernible qualitative differences in outcomes.

### Statistical analyses

The correction for multiple comparisons in Figure 1B was done through cluster statistics. Group comparison analyses for peak amplitudes and latencies were carried out using a two sample *t*-test.

## Results

### Source localization

As expected, the evoked responses from the pulses delivered onto the right index and middle fingers (Figure 1A) localized the primary (S1) somatosensory cortex of the contralateral hemisphere (Figure 1B). After correcting for multiple comparisons in time, using non-parametric cluster statistics (Maris and Oostenveld, 2007), none of the cluster was significant.

### No differences in amplitude or latency

The evoked responses in S1 in source space (Figures 1C,D) were analyzed for each participant. Amplitude and latency of the first peak of the evoked response were identified on a subject-by-subject basis, for each pulse. Thus, for each participant, we considered 4 peaks: M40 following the first pulse in the fast condition, M40 following the second pulse in the fast condition, M40 following the first pulse in the slow condition, and M40 following the second pulse in the slow condition. There was no difference in amplitude (Figure 2A) or latency (Figure 2B) between the groups for any of the 4 peaks (Figure 2). There was also no significant difference in the variability for amplitude or latency between the groups. Since the results showed no significant group difference even before any correction for multiple comparisons, there was no need for a correction.

### No correlation with behavioral scores

The amplitude and latency results were not correlated with the ADOS score, or with the touch score assessed using the sensory processing questionnaire, for any of the peaks. Grouping by touch score (normal or low) rather than by diagnosis also did not result in any significant group differences.

## Discussion

We found that both the amplitude and latencies of the evoked response to rapid sequences of tactile pulses were normal in ASD. The finding was contrary to our initial hypothesis, that rapid sensory processing deficits are prevalent in ASD across different sensory domains. These results suggest that tactile processing in ASD is not impacted by rapid presentations of stimuli.

The results are in line with a prior MEG study, that found no abnormalities in ASD in response to tactile pulses separated by 330 ms (Marco et al., 2012), slower than the 200 ms separation examined here. Notably, that same study did find abnormalities in the responses to far slower inter-pulse separation, at ~1300–1600 ms. Our fast interstimulus interval was considerably faster than the fast stimulus in Marco et al. and in line with the auditory rapid stimuli in Oram Cardy et al., which were spaced at 150 ms (Oram Cardy et al., 2005). Thus, it was not obvious prior to our study whether responses at 200 ms intervals would be normal in ASD, even given the Marco et al. study. Our study also included an intermediate interstimulus interval, at 700 ms. We did not see any group differences at that rate either. Therefore, the differences observed in Marco et al. for stimuli with a rate that is 1300 ms or slower likely emerge only at rates slower than about 1 Hz. It is worth pointing out that there are several important methodological differences between the two studies, most notably, our data was localized to the source using MNE, while the data in Marco et al. was analyzed in sensor space. In spite of various methodological differences, it is remarkable that both studies agree on the results at rates faster than 1 Hz. It is also worthwhile noting that another MEG study, where the rate of tactile pulse stimuli was not stated but was presumably fast, also did not find significant group differences in responses to single tactile pulses (Coskun et al., 2013). Thus, there seems to be general agreement between the very few studies that examined this question, that evoked responses to tactile pulses are not abnormal in ASD, at least for fast (1–5 Hz) stimulus rates.

This outcome diverges from findings in the auditory domain, and specifically responses to tone stimuli, which are the auditory domain parallel of tactile pulses. While evoked responses to certain tone stimuli are normal in ASD, latency, and or amplitude differences do exist under particular parameters, such as frequency of the tone being presented (Gage et al., 2003; Oram Cardy et al., 2004, 2008; Flagg et al., 2005; Roberts et al., 2010). These differences likely arise from the fundamental difference in specialization between the two domains. In particular, the auditory domain is optimized for speech processing, which relies heavily on feedback occurs on very fast time scales, generally faster than those associated with the somatosensory domains. At the same time, this outcome is in line with our prior work examining responses to vibrotactile stimuli in ASD, where we found abnormalities in functional connectivity, but normal evoked responses (Khan et al., 2015). These results align with other work from our group and others that measured abnormal functional connectivity in ASD, but normal evoked responses, in other sensory domains (e.g., Dinstein et al., 2011; Khan et al., 2013).

The outcomes need to be interpreted in the context of the limitations of the study. The timing parameters for the methodology of our pulse, short (200 ms) and long (700 ms) lie in between fast responses of previous studies showing group differences in auditory responses (Oram Cardy et al., 2005), and intermediate timings revealing no cortical effect in somatosensory responses (Marco et al., 2012). Thus, it is possible that even faster inter-stimulus intervals would result in findings more parallel to those in the auditory domain. Furthermore, as discussed on the methods, our analysis was restricted specifically to M40. While a preliminary investigation of the later components of the evoked response revealed no statistical group difference in those components either, it is possible that a paradigm more suitable for an investigation of later responses, would reveal differences in ASD in those components. The extension of the receptive field by simultaneous application of stimuli to the middle and right index fingers is also a limitation of the study, as it may have impacted temporal discrimination in S1 (Bolognini et al., 2010), in a way that would mask a slight temporal discrimination deficit in the ASD group. Thus, the existence of such a deficit cannot be fully excluded by the experimental design. Lastly, the small sample size is of course also a limitation of the study.

In summary, we observed normal evoked responses to tactile pulses in ASD, even when those were presented in very fast succession. These results suggest that the neural substrates that underlie the behaviorally observed somatosensory abnormalities in ASD are clearly more complex than those involved in generating the evoked response to simple pulses, regardless of their temporal separation.

## Author contributions

SG, SK, and TK designed research; SG, SK, KAG, and TK performed research; SG, SK, MSH, and TK analyzed data; and SG, SK, and TK wrote the paper.

## Funding

This work was supported by grants from the Nancy Lurie Marks Family Foundation (TK and SK), Autism Speaks (TK), The Simons Foundation (SFARI 239395, TK), The National Institute of Child Health and Development (R01HD073254, TK), The National Center for Research Resources (P41EB015896, MSH), National Institute for Biomedical Imaging and Bioengineering (5R01EB009048, MSH), and the Cognitive Rhythms Collaborative: A Discovery Network (NFS 1042134, MSH).

### Conflict of interest statement

The authors declare that the research was conducted in the absence of any commercial or financial relationships that could be construed as a potential conflict of interest.
